# Interventional Radiotherapy (Brachytherapy) Combined with Systemic Treatment—The Influence of RAS Gene Mutations and Combined Therapy on the Results and Toxicity of Colorectal Cancer Liver Metastases

**DOI:** 10.3390/cancers17213530

**Published:** 2025-10-31

**Authors:** Paweł Cisek, Aleksandra Kozłowska, Ludmiła Grzybowska-Szatkowska

**Affiliations:** Department of Radiotherapy, Medical University of Lublin, 20-081 Lublin, Poland; akozlowska@usk1.pl (A.K.); ludgr@poczta.onet.pl (L.G.-S.)

**Keywords:** *RAS* mutation, brachytherapy, liver metastases, interventional radiotherapy, anti-EGFR, anti-VEGFR

## Abstract

**Simple Summary:**

The study aimed to evaluate the outcomes and complications of HDR brachytherapy for colorectal liver metastases, taking into account RAS gene mutation status, targeted therapy and the number of chemotherapy regimens. This retrospective study analysed 142 patients with oligoprogressive liver metastases who were treated with HDR brachytherapy between 2015 and 2022 without a change in systemic therapy. RAS mutations, treatment lines, PFS, OS, LC, radiological response, hepatotoxicity and bleeding risk were assessed. KRAS/NRAS mutation were found to significantly worsen PFS, OS and LC, particularly in the third and fourth treatment lines. The best outcomes were seen in RAS wild-type patients, who were treated with EGFR inhibitors in the first line and trifluridine/tipiracil in the third line. The most effective tumour control and volume reduction were observed in the first three treatment lines. Treatment was generally well tolerated, with minor changes to albumin levels in later lines. RAS mutations are associated with a poorer prognosis across treatment lines. HDR brachytherapy is most beneficial in early treatment lines and in RAS wild-type patients. Combining brachytherapy with systemic therapy is safe, regardless of the regimen used.

**Abstract:**

Introduction: The aim of the study was to analyse the results and potential complications of local treatment with HDR (high dose-rate) brachytherapy of liver metastases from colorectal cancer, depending on the targeted therapy used and considering *RAS* gene mutation and chemotherapy in individual treatment lines. Material and methods: The study included 142 patients with oligoprogressive liver metastases who underwent HDR brachytherapy without changing the line of treatment, based on a retrospective analysis of 270 patients treated between 2015 and 2022. The impact of *RAS* gene mutations, lines of chemotherapy depending on the treatment regimens used, PFS (progression free survival), OS (overall survival), LC (local control) and the degree of radiological response were analysed. The impact of these drugs on hepatotoxicity and the risk of haemorrhagic complications was also analysed. Results: The presence of mutations in *KRAS/NRAS* genes (exons 2, 3, 4) had a statistically significant impact on PFS in the first, third and fourth lines of treatment, and on OS and LC in the third and fourth lines of treatment. In the third and fourth lines of treatment, patients with a mutation in the *RAS* gene had a poorer radiological response to treatment regardless of the chemotherapy used. PFS, OS and LC differed depending on the line of treatment and amounted to 17.5, 11, 8.5, 6 and 4 months, 27, 19, 13, 11 and 11 months, and 27, 19, 11, 6 and 6 months, respectively. The greatest benefit in terms of PFS was achieved by patients treated with first-line chemotherapy combined with epidermal growth factor receptor (EGFR) inhibitors, in the absence of *RAS* gene mutations. In the third line, the greatest benefit was achieved by patients treated with trifluridine/tipiracil in the absence of *RAS* gene mutations. The greatest percentage reduction in the volume of treated lesions and the highest percentage of control were observed in the first three lines of treatment. The toxicity of the treatment was low; only in the third and fourth lines of treatment were differences in the decrease in albumin levels found depending on the type of treatment used. Conclusions: A mutation in the *RAS* genes worsens the prognosis, regardless of the line of treatment and the systemic treatment used. The greatest benefit from brachytherapy is seen in patients in the first three lines of treatment without *RAS* mutations, treated with anti-EGFR chemotherapy in the first line and trifluridine/tipiracil in the third line. Combining brachytherapy of liver metastases with systemic treatment is safe, regardless of the systemic treatment used.

## 1. Introduction

Metastases of colorectal cancer to the liver is one of the greatest challenges in modern oncology. Effective treatment often requires a combination of systemic therapies and local methods. The gold standard for local therapy is the surgical removal of metastases, which can achieve a 5-year survival rate of 25–40%. However, only around 25% of patients are eligible for primary resection, and a further 5% for secondary resection [1]. When resection is not possible, alternative local treatments are available, including thermal ablation (e.g., radiofrequency ablation (RFA) and laser interstitial thermal therapy (LITT), transarterial chemoembolisation (TACE), yttrium-90 radioembolisation (Y-90 RE) and stereotactic radiotherapy (SBRT)). However, many of these techniques are limited by factors such as the size, number and location of metastases, as well as the presence of anatomical structures that constitute a barrier to the safe use of a given method [2]. Although SBRT is a widely recognised non-invasive technique, it is limited by the dose tolerance of adjacent organs, especially in the case of numerous large tumours [3,4]. In this context, high-dose brachytherapy (HDR) appears to be a promising alternative. Unlike SBRT, in brachytherapy the radiation source is placed inside the tumour. Thanks to the rapidly decreasing dose distribution resulting from the interaction of the radiation source, a very high dose can be delivered directly to the tumour with minimal exposure of neighbouring tissues. Other advantages include that planning and treatment times are short, the method is independent of the patient’s position and the treatment has minimal impact on organ mobility. Disadvantages include the invasiveness of the method, the need for properly trained personnel and the requirement of a specialised imaging system. Although there is limited high-quality data for brachytherapy and no reports comparing it with SBRT, dosimetric data suggest that brachytherapy offers advantages with respect to critical organ doses [5]. To maintain control of metastatic disease at the oligoprogression stage, systemic treatment is an integral part of management [6]. However, data on the optimal combination of ablation techniques using SBRT with systemic treatment are scarce [7].

A mutation in the *RAS* gene is common in colorectal cancer and directly influences treatment. Most data on the impact of RAS mutations on prognosis and radiotherapy outcomes are available. *KRAS* mutations lead to increased cell proliferation, resistance to apoptosis and increased capacity for DNA repair, including the repair of damage induced by ionising radiation [8]. Currently, the data is limited to radiotherapy and does not consider systemic treatment. There are no data on combining systemic treatment with brachytherapy in regimens involving chemotherapy and targeted therapy based on *RAS* gene mutations. This study aimed to analyse the treatment outcomes of patients with oligoprogressive liver metastases who underwent brachytherapy in combination with systemic therapy, depending on *RAS* mutation status and the type of systemic therapy received. The next objective was to ascertain the safety of combining brachytherapy with systemic therapy and identify the potential complications of this treatment.

## 2. Materials and Methods

### 2.1. Patient Selection Criteria

This study is a part of retrospective analysis of 270 patients with liver metastases from colorectal cancer who were treated with HDR brachytherapy at the Brachytherapy Department of the Lublin Cancer Centre between 2015 and 2022. Of these patients, only those with recurrent or induced oligoprogression (202 patients) were selected. Oligoprogression was defined as progression of no more than five metastases with no progression (stabilisation or regression) of the remaining metastases.

**Figure 1:**
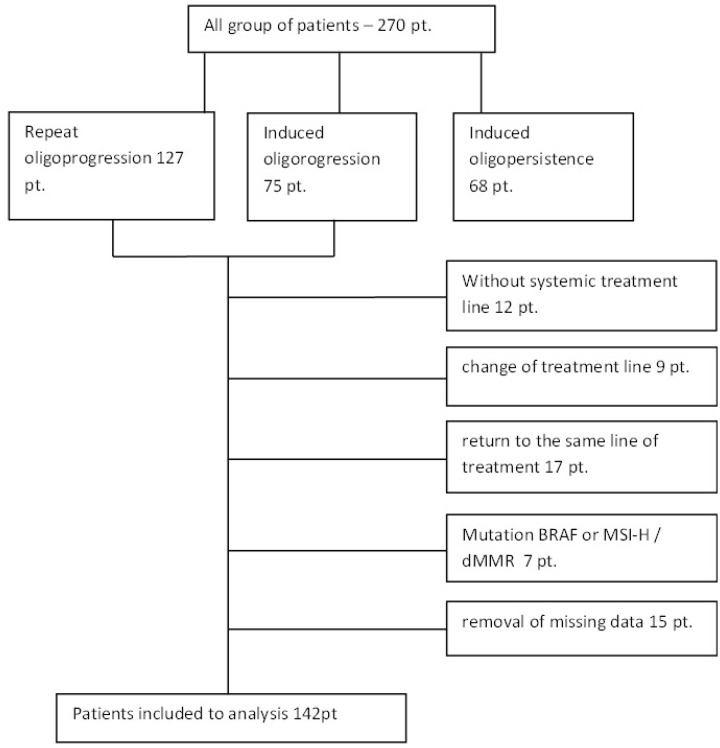
Patient selection.

Due to the minimal percentage of patients with mutations in the *BRAF* or MSI-H/dMMR (microsatellite instability-high/deficient mismatch repair) genes, as well as different prognoses and other options for systemic treatment, this group of patients was excluded from the study. Following the removal of patients who had changed or resumed systemic treatment, those with a *BRAF* mutation or MSI-H/dMMR and those with incomplete data on systemic treatment or a change in treatment line, 142 patients who had received the same systemic treatment after oligoprogression were selected (see Figure 1).

The study included patients with a World Health Organization (WHO) performance status of 2 or less, who were haematologically competent (haemoglobin (HGB) > 8 mg/dL, white blood cell (WBC) count > 2000/mm^3^, neutrophil (NEU) count > 1500/mm^3^, platelet (PLT) count > 50,000/mm^3^), with properly functioning livers and kidneys (alanine transaminase (ALT), aspartate transaminase (AST) and total bilirubin (BIL) levels < 2.5× the upper limit of normal, creatinine level < 2 mg/dL), with metastases of a maximum diameter of 15 cm and a maximum of five metastases. Patients were excluded if metastases were not resectable or if there was an inability or contraindication to liver applicator placement.

### 2.2. Cohort Characteristics and Treatments

All patients had histopathologically confirmed colorectal cancer, and a radiological and/or histopathological examination confirmed liver metastasis. The *RAS* gene mutation status (*KRAS/NRAS*, exons 2, 3 and 4) was used to predict systemic treatment in all patients. Based on this, patients were eligible for subsequent lines of systemic treatment. First-line treatment consisted of regimens based on fluoropyrimidine derivatives with either oxaliplatin or irinotecan, alongside an epidermal growth factor receptor (EGFR) inhibitor (wild type of the *RAS* gene) or a vascular endothelial growth factor (VEGF) inhibitor. Second-line treatment also included a regimen based on fluoropyrimidine with either oxaliplatin or irinotecan, depending on the drug used in the first line, and an anti-VEGFR drug. For the third and subsequent lines of treatment, trifluridine/tipiracil, regorafenib, EGFR inhibitors, other fluoropyrimidine derivatives or irinotecan were used. Brachytherapy was performed between systemic treatment cycles so that the interval did not exceed one week. For VEGFR inhibitors (bevacizumab and aflibercept) and tyrosine kinase inhibitors that act on the VEGFR receptor (regorafenib), the interval between anti-angiogenic treatment and brachytherapy was at least two weeks. If chemotherapy with an anti-VEGFR drug was scheduled during this period, only chemotherapy was administered. A multidisciplinary team performed the qualification for treatment. Patients were selected for one of the following local treatment options: surgery, chemoembolisation, stereotactic body radiotherapy (SBRT) or brachytherapy. The following factors were considered: general condition, disease progression, number and volume of metastases, technical feasibility, systemic treatment regimen and patient preferences. The characteristics of the patients are presented in Table 1. The systemic treatment of patients is presented in Table 2.

The application was performed under continuous tomographic imaging. Doses in the range of 15–25 Gy were used, with the main limitation being the dose to two-thirds of the liver parenchyma after subtracting the metastases, which did not exceed 5 Gy. Technical and dosimetric details can be found in previous papers [5,9,10].

### 2.3. Analysis of Mutations in the RAS Genes

The study was performed on a tissue block from either a primary or a metastatic tumour in the liver. QPCR (quantitative polymerase chain reaction) was used alongside the QIAamp DSP DNA FFPE Tissue Kit (Qiagen, Dűsseldorf, Germany), which is designed for silica-based DNA purification from formalin-fixed, paraffin-embedded tissue. Gene mutation detection was performed using three kits: the *KRAS* Mutation Analysis Kit CE-IVD (Entrogen, Woodland Hills, CA, USA) for 18 mutations within codons 12, 13, 61, 117 and 146 in exons 2, 3 and 4; the *NRAS* Mutation Analysis Kit CE-IVD (Entrogen, Woodland Hills, CA, USA) for 10 mutations within the same codons and exons; and the *RAS* c59/117 Mutation Detection Kit CE-IVD (Entrogen, Woodland Hills, CA, USA) for 1 mutation within codon 59 in the *KRAS* gene (exon 3) and 2 mutations within codons 59 and 117 in the *NRAS* gene (exons 3 and 4). These kits were used on a Cobas z480 instrument (Roche, Basel, Switzerland). The results were analysed using LightCycler 480 SW software ver. 1.5.1 (Roche, Basel, Switzerland). The analysis of the frequency of individual mutations is presented in Table 3. No *KRAS* G12C mutations were detected in the study group.

### 2.4. Follow Up

During the post-treatment period, patients underwent periodic imaging examinations, including computed tomography or magnetic resonance imaging, four to six times a year. Response to treatment was assessed using RECIST 1.0 criteria. Due to difficulties in interpreting the CT images, an MRI scan was also performed on the patients. Due to the risk of pseudoprogression in the first six weeks after irradiation, the response to treatment was assessed using CT scans at least 12 weeks after brachytherapy (following two consecutive scans at four-weekly intervals). Patients were assigned to the appropriate response category according to RECIST criteria based on changes in the size of metastases visible on CT images. If an MRI scan was performed, changes in size and functional sequences (DWI) were both evaluated to assess the response. Patients were monitored throughout the treatment period until there were no more therapeutic options left or unacceptable toxicity was reached. Toxicity was assessed on the CTCAE scale version 5.0.

### 2.5. Statistical Analysis

Survival analysis was performed using the Kaplan–Meier method. The Log-rank test was used to analyse the factors affecting local control (LC), progression-free survival (PFS) and overall survival (OS). LC was defined as the time from brachytherapy to the progression of the brachytherapy-treated metastasis as seen on imaging scans (CT or MRI). PFS was defined as the time from brachytherapy to the progression of any metastasis (whether or not it had been treated with brachytherapy) or the appearance of a new metastasis. OS was defined as the time from brachytherapy to patient death. The Mann–Whitney U non-parametric test was used to examine the relationship between independent variables or to compare two groups of patients, while the Kruskal–Wallis test was used for multiple group comparisons. To compare the frequency of analysed categories depending on studied parameters, a non-parametric chi-squared test was used for qualitative variables. A *p*-value of less than 0.05 was considered to indicate a statistically significant difference. Statistical analysis was performed using Statistica version 13 software (Statsoft, OK, Tulsa, USA).

All procedures performed in studies involving human participants were in accordance with the ethical standards of the institutional research committee and with the 1964 Helsinki Declaration and its later amendments or comparable ethical standards. The study was approved by the Lublin Medical Chamber no. LIL-KB-20/2014. Written consent was obtained from each patient.

## 3. Results

### 3.1. The Impact of the Line of Systemic Treatment

**Figure 2:**
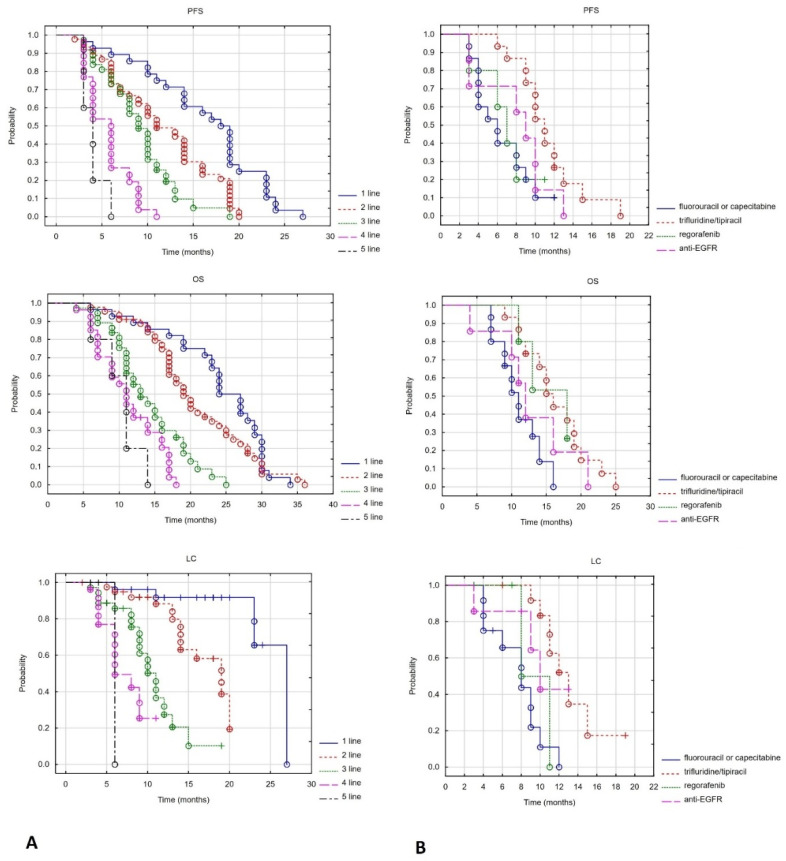
PFS, OS and LC depend on the line of systemic treatment (**A**) and on the type of third-line systemic treatment (**B**).

In the analysed group of patients, the median progression-free survival (PFS) was 9.5 months, the median local control (LC) was 15 months and the median overall survival (OS) was 17 months. The 12-month PFS LC and OS were 37% 63% and 78%, respectively. A statistically significant reduction in 12-month PFS, LC and OS was observed with subsequent lines of systemic treatment. The data are presented in Table 4 and Figure 2A.

### 3.2. The Impact of RAS Mutation

**Figure 3:**
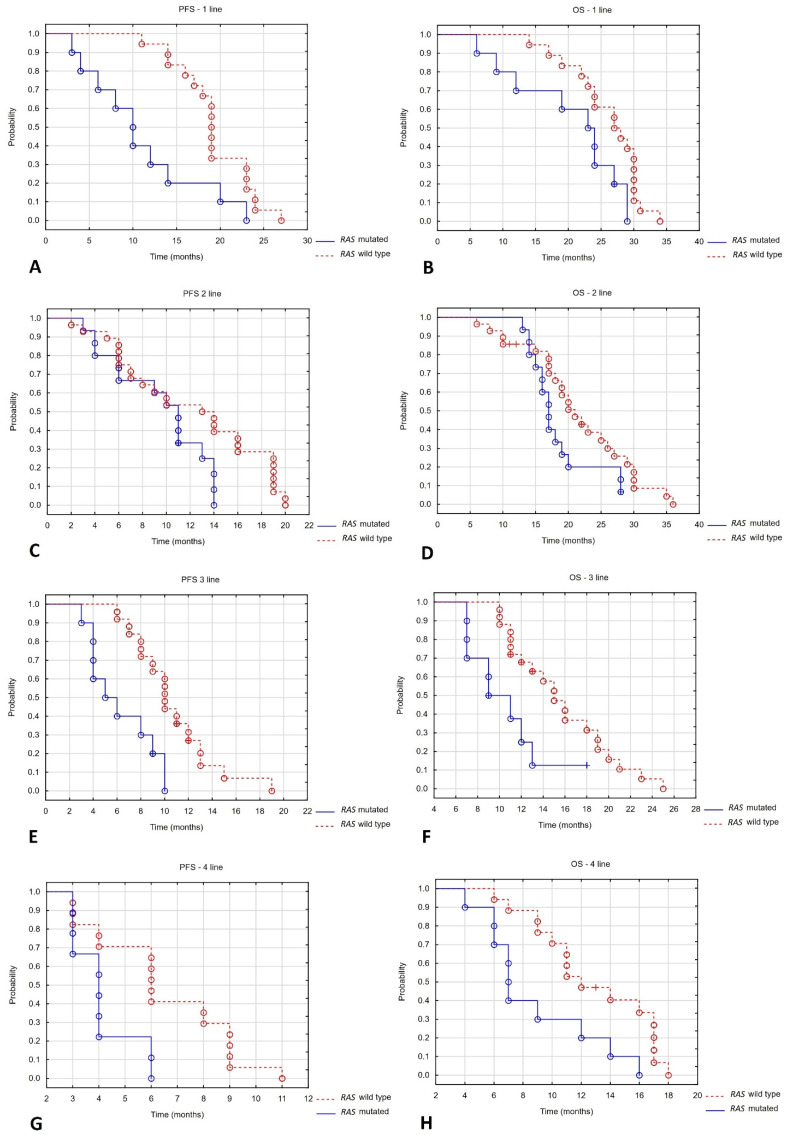
PFS and OS depending on systemic treatment line. (**A**) PFS-1 line; (**B**) OS-1 line; (**C**) PFS-2 line; (**D**) OS-2 line; (**E**) PFS-3 line; (**F**) OS-3 line; (**G**) PFS-4 line; (**H**) OS-4 line.

In first-line treatment, a statistically significant difference in PFS was found depending on *RAS* gene mutation status, with patients with *RAS* gene mutations having a poorer prognosis. No statistically significant differences in OS were found, although the result was close to statistical significance. No statistically significant differences in LC were observed. Similarly, no statistically significant differences were observed in tumour volume reduction or RECIST response rate. Similarly, differences close to statistical significance were found in terms of PFS in the second-line treatment. However, in the case of OS, the result was not statistically significant. No statistically significant differences were found in LC, tumour volume reduction or RECIST response rate depending on the type of systemic treatment combined with brachytherapy. In the third-line treatment, statistically significant differences were observed in terms of PFS, LC and OS. A marginally statistically significant difference was also observed in the percentage of tumour volume reduction and RECIST response rate. Among patients with mutations, four out of ten (40%) progressed, compared to two out of twenty-five (8%) in the mutation group. Similarly, statistically significant differences were found in PFS, LC and OS in the fourth line of treatment. The differences in percentage tumour volume reduction and RECIST scale responses were marginally statistically significant. Progression was observed in seven out of ten (70%) patients with the mutation, compared to three out of seventeen (18%) without the mutation. No analysis was performed in the fifth line of treatment due to the small number of patients. See Table 5 and Figure 3.

### 3.3. First-Line Treatment

**Figure 4:**
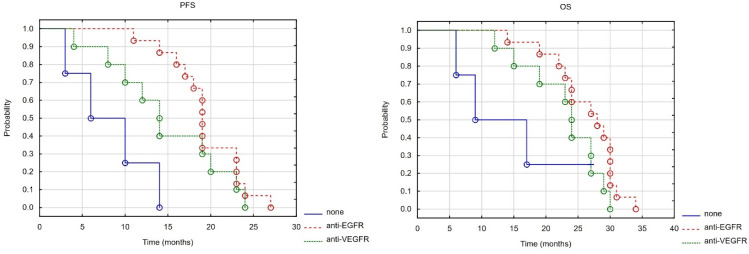
PFS and OS depending on targeted therapy (anti-EGFR and anti-VEGFR) received by patients who underwent combined systemic treatment with brachytherapy.

No statistically significant differences were found in the effectiveness of first-line treatment, whether irinotecan- or oxaliplatin-based. Median PFS was 18.5 months in the irinotecan group and 19 months in the oxaliplatin group (Log-rank test value = 0.30, *p* = 0.975). Median OS was 27 months in the irinotecan group and 29 months in the oxaliplatin group (Log-rank test value = 0.650, *p* = 0.515). Median LC was not reached in the irinotecan group and was 23 months in the oxaliplatin group (Log-rank test value = −0.237, *p* = 0.812). In the entire group of patients undergoing first-line systemic treatment, there were statistically significant differences in PFS depending on whether combination chemotherapy was used alongside anti-EGFR and anti-VEGFR drugs, or whether no combination drugs were used (Log-rank test value = 9.030, *p* = 0.011). Median PFS was 19, 14 and 10 months in the groups of patients treated with fluoropyrimidine derivatives (5-fluorouracil or capecitabine), EGFR and VEGFR receptor inhibitors and fluoropyrimidine derivatives alone, respectively. There were statistically significant differences in median PFS between patients treated with fluoropyrimidine derivatives and EGFR inhibitors, and fluoropyrimidine derivatives alone (Log-rank test value = 2.238, *p* = 0.025). However, no differences were found between fluorouracil (FU) with VEGF inhibitors and FU alone (Log-rank test value = 1.431, *p* = 0.152), or between FU with EGFR inhibitors and FU with VEGF inhibitors (Log-rank test value = −1.304, *p* = 0.192). Although the result was close to statistical significance, no statistically significant differences in OS were found in the entire group (Log-rank test value = 4.867, *p* = 0.088). Median OS was 28, 24 and 17 months in the groups of patients treated with EGFR, VEGFR inhibitors and none of these drugs, respectively. Nevertheless, no significant differences were observed between FU and EGFR inhibitors (Log-rank test value = 1.272, *p* = 0.203), FU with VEGFR inhibitors and FU alone (Log-rank test value = 0.702, *p* = 0.482), or between FU with EGFR inhibitors and FU with VEGFR inhibitors (Log-rank test value = −1.604, *p* = 0.109). No statistically significant differences in terms of local control were found in the entire group depending on the use of combination chemotherapy with anti-EGFR and anti-VEGFR drugs, or the use of neither of these drugs (Log-rank test value = −0.228, *p* = 0.819) (see Figure 4). 

### 3.4. Second-Line Treatment

In the second line of treatment, no statistically significant differences were found between the drugs used in PFS (Log-rank test value = 0.941, *p* = 0.625). Similarly, no differences were observed between the combination of FU with either oxaliplatin or irinotecan (Log-rank test value = −0.563, *p* = 0.573). The median PFS for FU, FU combined with oxaliplatin and FU combined with irinotecan was 11, 9 and 13 months, respectively. There were no statistically significant differences in OS (Log-rank test value = 3.561, *p* = 0.168), although a numerically significant difference in median OS was observed between FU and FU combined with either oxaliplatin or irinotecan (median OS was 14, 19 and 21 months, respectively). There were no statistically significant differences between FU combined with oxaliplatin or irinotecan (Log-rank test value = 0.524, *p* = 0.599), between FU alone and FU combined with oxaliplatin (Log-rank test value = 1.431, *p* = 0.152), or between FU alone and FU combined with irinotecan (Log-rank test value = −1.517, *p* = 0.129). However, given the small number of patients who received FU alone, the differences between FU alone and FU in combination with either oxaliplatin or irinotecan are evident and close to statistical significance. Similarly, no statistically significant differences were found in terms of LC (Log-rank test value = 2.041, *p* = 0.360). Median LC for FU alone, FU with oxaliplatin and FU with irinotecan was 19, 19, and 14 months, respectively. There were no differences in LC between the combinations of FU with either oxaliplatin or irinotecan (Log-rank test value = 0.147, *p* = 0.882), between FU alone and the combination of FU with irinotecan (Log-rank test value = 1.364, *p* = 0.172), or between FU alone and the combination of FU with oxaliplatin (Log-rank test value = 1.715, *p* = 0.086). There were no statistically significant differences in PFS between patients who received chemotherapy without a VEGFR inhibitor and those who received chemotherapy in combination with a VEGFR inhibitor (Log-rank test value = 0.109, *p* = 0.913). Median PFS was 11 and 13 months in patients who received chemotherapy without a VEGFR inhibitor and in patients who received chemotherapy in combination with a VEGFR inhibitor, respectively. Similarly, no statistically significant differences in OS were observed between the two groups (Log-rank test value = −0.128, *p* = 0.898). Median OS was 19 and 20 months in patients who received chemotherapy without a VEGFR inhibitor and in patients who received chemotherapy in combination with a VEGFR inhibitor, respectively. No statistically significant differences in LC were observed (Log-rank test value = −0.320, *p* = 0.749).

### 3.5. Third-Line Treatment

In the third line of treatment, statistically significant differences in PFS were found between patients receiving only traditional fluoropyrimidine derivatives (fluorouracil and capecitabine) and those receiving trifluridine/tipiracil, regorafenib or EGFR inhibitors (Log-rank test value = 11.337, *p* = 0.01). The median PFS for patients receiving fluorouracil or capecitabine, trifluridine/tipiracil, regorafenib or EGFR inhibitors was 6, 11, 7 and 9 months, respectively. Statistically significant differences were only found when comparing fluorouracil or capecitabine with trifluridine/tipiracil (Log-rank test value = 2.559, *p* = 0.011). Statistically significant differences in OS were also found between patients receiving only traditional fluoropyrimidine derivatives (fluorouracil or capecitabine) and those receiving trifluridine/tipiracil, regorafenib or EGFR inhibitors (Log-rank test value = 8.744, *p* = 0.033). The median OS for patients receiving fluorouracil or capecitabine, trifluridine/tipiracil, regorafenib or EGFR inhibitors was 10, 15, 13 and 11 months, respectively. Only statistically significant differences were found between patients receiving fluorouracil or capecitabine and those receiving trifluridine/tipiracil (Log-rank test value = 2.196, *p* = 0.028). Statistically significant differences were also found in terms of LC (Log-rank test value = 13.311, *p* = 0.004). The median LC for patients receiving fluorouracil or capecitabine, trifluridine/tipiracil, regorafenib and EGFR inhibitors was 8, 12, 10 and 8 months, respectively. No statistically significant differences were found in a direct comparison between the above drugs (*p* > 0.05). See Figure 2B.

### 3.6. Fourth-Line Treatment

In the fourth line, no statistically significant differences in PFS were found between patients receiving fluorouracil/capecitabine, trifluridine/tipiracil, regorafenib/irinotecan or brachytherapy (Log-rank test value = 6.910, *p* = 0.075). The median PFS was 4, 8, 6.5 and 6 months, respectively. Similarly, no statistically significant differences in OS were found between patients receiving fluorouracil/capecitabine, trifluridine/tipiracil, regorafenib or irinotecan (Log-rank test value = 6.517, *p* = 0.089). The median OS was 4, 17, 11 and 9 months, respectively. No statistically significant differences in LC were found between patients receiving fluorouracil/capecitabine, trifluridine/tipiracil, regorafenib or irinotecan (Log-rank test value = 6.091, *p* = 0.108). The median LC for trifluridine/tipiracil was not reached; for fluorouracil/capecitabine, regorafenib and irinotecan it was 6, 6 and 9 months, respectively.

### 3.7. Tumour Volume Reduction and Response Rate According to RECIST

**Figure 5:**
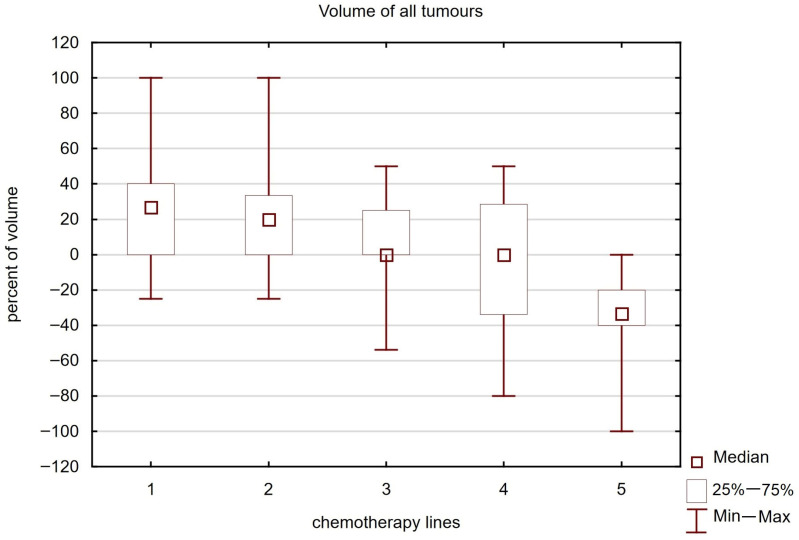
Reduction in tumour volume depending on systemic treatment line with brachytherapy.

A statistically significant difference in the percentage of tumour reduction was found depending on the line of treatment (Kruskal–Wallis test value = 13.218, *p* = 0.010). Most patients experienced a reduction in tumour volume in the first to third lines of treatment (up to 40%, 34% and 25%, respectively). In the fourth line of treatment, half of the patients responded with a reduction in tumour mass. In the fifth line of treatment, most patients experienced tumour progression (see Figure 5). This was undoubtedly related to the brachytherapy dose. In the first line of treatment, 25 Gy was administered to 13/28 patients (46%), in the second line to 20/45 patients (44%) and in the third line to 15/37 patients (40%). In the fourth and fifth lines, a dose of 25 Gy was administered to 4/27 (15%) and 0/5 (0%) patients, respectively. These differences were statistically significant (Chi-square test value = 15.616, *p* = 0.048). However, no statistically significant differences were found in terms of the volume of treated metastases (Kruskal–Wallis test value = 5.298, *p* = 0.258).

No statistically significant differences were found in the percentage of tumour reduction depending on the type of chemotherapy in the first line of treatment (Kruskal–Wallis test value = 0.235, *p* = 0.814); the type of targeted therapy in the first line (U Mann–Whitney test value = 0.432, *p* = 0.805); the type of chemotherapy in the second line of treatment (Kruskal–Wallis test value = 2.817, *p* = 0.244); the use of VEGFR inhibitors in the second line (U Mann–Whitney test value = 1.255, *p* = 0.209); the type of chemotherapy in the third line of treatment (Kruskal–Wallis test value = 5.4, *p* = 0.145) and the type of chemotherapy in the fourth line of treatment (Kruskal–Wallis test value = 7.314). *p* = 0.062). Based on the RECIST assessment criteria, a statistically significant difference in response to treatment was found depending on the line of systemic treatment used (Chi-square test value = 30.158, *p* = 0.002). The respective percentages of objective responses in subsequent lines of treatment were 96% (CR = 7%, PR = 39%, SD = 50%), 93% (CR = 11%, PR = 22%, SD = 60%), 81% (CR = 0%, PR = 24%, SD = 57%), 63% (CR = 0%, PR = 23%, SD = 30%) and 40% (CR = 0%, PR = 0%, SD = 40%). No statistically significant differences in response to treatment were found depending on the type of chemotherapy in the first line of treatment (Chi-square test value = 1.238, *p* = 0.743); the type of targeted therapy in the first line of treatment (Chi-square test value = 11.157, *p* = 0.083); the type of chemotherapy in the second line of treatment (Chi-square test value = 5.380, *p* = 0.496); the use of VEGFR inhibitors in the second line of treatment (Chi-square test value = 3.695, *p* = 0.296); the type of chemotherapy in the third line of treatment (Chi-square test value = 7.111, *p* = 0.625) and the type of chemotherapy in the fourth line of treatment (Chi-square test value = 12.181, *p* = 0.203).

### 3.8. Toxicity Analysis

**Figure 6:**
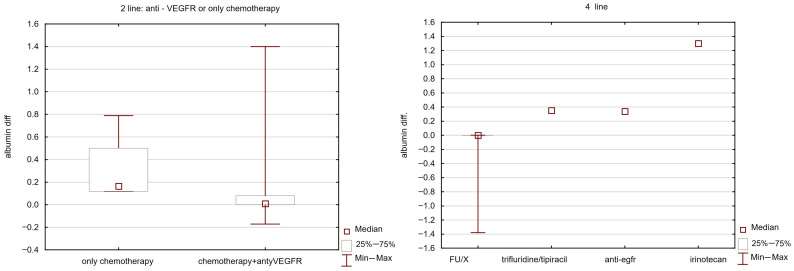
Depending on the treatment used, there is a decrease in albumin levels (g/dl) at systemic lines 2 (**left**) and 4 (**right**).

The clinical features of liver damage were analysed in terms of the following commonly used liver function tests: total bilirubin, aspartate aminotranspherase (AST), alanine aminotranspherase (ALT), albumin level (ALB) and prothrombin time (PT). Grade 1 hyperbilirubinaemia was observed in 7% of patients (9/133), and grade 2 observed in 3% (3/133). Grade 1 toxicity associated with elevated ALT levels was observed in 15 patients (11%), while grade 2 toxicity occurred in 3 patients (2%). Grade 1 toxicity associated with increased AST levels was observed in 22 patients (17%), and grade 2 toxicity was observed in 4 patients (3%). No statistically significant differences in hepatic toxicity were found depending on the line of systemic treatment. However, a statistically significant difference in the decrease in albumin levels was found depending on the addition of a VEGFR inhibitor. In the absence of a VEGFR inhibitor, the median maximum decrease in albumin levels was 0.19 g/dL; with the addition of a VEGFR inhibitor, it was 0.01 g/dL. A statistically significant difference was also found in systemic treatment in the fourth line. In patients treated with irinotecan, the median maximum decrease in albumin levels was 1.3 g/dL, whereas with other drugs it ranged from 0 to 0.39 g/dL. No statistically significant differences were found between individual drugs in other lines of treatment. See Figure 6 and Table 6. 

In the analysed group of patients, a maximum decrease in haemoglobin levels of one grade occurred in 31 out of 118 patients (26%), a decrease of two grades occurred in 13 out of 118 patients (11%) and a decrease of three grades occurred in 1 out of 118 patients (<1%). A decrease in platelet count by one grade occurred in 27 out of 116 patients (23%) and by two grades in four out of 116 patients (4%). Visible clinically apparent bleeding in imaging tests occurred at grade 1 in 11/141 patients (8%) and at grade 2 in three patients (2%). No clinical bleeding of grade 3 or higher was observed. No statistically significant differences were found in HGB, PLT or clinically apparent bleeding depending on the type or line of systemic treatment; see Table 7. No other complications related to the procedure occurred, such as infection or pneumothorax.

## 4. Discussion

Despite stereotactic radiotherapy being widely used for the local treatment of liver cancer, HDR brachytherapy was used for the analysed group of patients. The high efficacy of this method has been confirmed to date in both liver metastases and primary liver cancer, including in a phase II study [11,12,13]. While there are no data on a direct comparison of brachytherapy and stereotactic radiotherapy, dosimetric studies suggest that a high dose can be administered to the centre of the tumour—a potentially radiation-resistant and poorly vascularised area [14,15]. Lower doses in critical organs also increase the safety of the therapy, and brachytherapy is not significantly associated with haemorrhagic complications in experienced centres [16]. Furthermore, despite SBRT itself being non-invasive, it is often necessary to insert fiducial markers, which are also associated with surgical complications [17]. The data from the study group comes from one of Poland’s largest oncology centres, where many years of experience with this method have been gained with a large group of patients [15,18].

Mutations in *RAS* genes (*NRAS* and *KRAS*) play a significant role in the pathogenesis of colorectal cancer. They constitute an independent prognostic factor and determine resistance to epidermal growth factor receptor (EGFR) inhibitors such as cetuximab and panitumumab [19]. *KRAS* mutations lead to the constitutive activation of the *RAS*–RAF–MEK–ERK signalling pathway. This results in increased cell proliferation, resistance to apoptosis, and an enhanced capacity for DNA repair, including the repair of damage induced by ionising radiation. In vitro models have shown that cells with *KRAS* mutations are less sensitive to radiotherapy than their wild-type counterparts [8,20]. However, some studies in unselected patient groups indicate that *RAS* gene mutations have no negative impact on the prognosis of patients undergoing SBRT [21]. A prospective multicentre cohort study investigating the effect of SBRT on oligometastatic colorectal cancer in various locations (liver, lymph nodes, lungs and bone) showed no difference in local control between wild-type and *KRAS*-mutated cases (*p* = 0.63). However, there was an improvement in PFS (hazard ratio (HR) 0.42, 95% confidence interval (CI): 0.27; *p* = 0.02) [22]. Other studies, in turn, indicate a predictive effect of *KRAS* on LC compared to wild-type *KRAS* tumours. Patients with *KRAS* mutations have worse LC (43%) compared to those with wild-type *KRAS* (72%; *p* < 0.02) [23]. The study by Jethwa et al. also demonstrated an increased risk of local failure in patients with a *KRAS* mutation in combination with a *TP53* mutation (HR 4.5; 95% CI: 1.1–18.7; *p* = 0.044) [24].

The results of the study of a group of patients undergoing brachytherapy for liver metastases indicate that *RAS* gene mutations have a negative impact on brachytherapy outcomes in most lines of treatment in a strictly selected group of patients. The main differences were found in PFS, with only the second line showing no statistically significant results. The most evident differences in LC, OS, tumour reduction and response to treatment were found in the third and fourth lines of treatment. The higher efficacy of anti-EGFR therapy also indirectly suggests a better prognosis for patients with the wild-type *RAS* gene. Therefore, there are no evident differences in prognosis in the second line of treatment, where anti-EGFR inhibitors were not used.

The use of radiotherapy due to oligoprogression during systemic treatment is common in colorectal cancer metastases and other cancers. In one study, the use of SBRT enabled local control in 70–100% of cases within a year [25]. In a study by Merino Lara et al. [26], 21.5% of patients with lung cancer required initiation of or change in systemic treatment within one year. However, the use of brachytherapy in cases of colorectal cancer metastases due to oligoprogression while maintaining the current line of treatment has not yet been studied. The 12-month local control (12 m LC), overall survival (12 m OS) and progression-free survival (12 m PFS) rates in the analysed group of patients were 63%, 78% and 37%, respectively. These results are similar to those reported in the literature for unselected groups of patients with various cancers [27]. In colorectal cancer, 12- and 15-month LC were 88% and 75%, respectively, but these studies do not provide data on maintaining the same treatment regimen for all patients [28,29]. It is difficult to compare the local control results of brachytherapy with those of SBRT. LC with brachytherapy is slightly worse; however, patients undergoing brachytherapy generally have larger metastases in diameter (the median volume of all treated metastases is 125 mL (range 1.1–1370 mL)), compared to data from the literature on SBRT (median 40 mL, range 1.6–877 mL) [30]. Most of the data on SBRT also lacks information on the impact of systemic treatment.

The study demonstrated the undeniable impact of systemic treatment lines on prognosis. PFS, OS, LC, tumour regression rate and response to treatment were all associated with systemic treatment lines. Patients in earlier lines of treatment experienced longer LC, OS and PFS. In later lines of treatment, lower tumour volume regression and a lower percentage of disease control were observed. This was probably related to the intensity of systemic treatment and the radiotherapy dose; however, there was no correlation with the volume of metastases. Lower doses were used in patients in later treatment lines, due to their general condition and concerns about liver function. These data indicate that brachytherapy may offer the greatest benefit when integrated earlier in the management of metastatic colorectal cancer rather than being reserved as a last-line option. There are no data indicating similar comparisons in such a group of patients. In the study by He et al. [31], SBRT was used in cases of oligoprogression of colorectal cancer metastases to the liver or lungs. No effect of the chemotherapy line used was demonstrated, but the number of patients undergoing concurrent chemotherapy was small and mostly comprised the first two lines of treatment. In the study by Ji et al. [32], it was found that more than one previous line of systemic treatment was associated with a statistically significant higher risk of local progression and first event distant relapse in a group of 94 patients. A poorer prognosis in subsequent lines of treatment affects the determination of the shield volume and increases resistance to chemotherapy [33]. For this reason, some authors suggest that local control deteriorates after previous chemotherapy [34].

Data from the literature suggest that both EGFR and VEGFR inhibitors have radiosensitising effects. EGFR inhibitors increase tumour control by sensitising it to radiation, inhibiting DNA repair and potentially destroying cancer cells [35]. The best-known application is cetuximab in combination with radiotherapy for head and neck cancers, although the role of cetuximab as a radiosensitiser has been debated in recent years [36]. Much less data is available on the combination of radiotherapy with VEGFR inhibitors, but the theoretical premise of inducing vascular normalisation to improve tumour perfusion and oxygenation increases the effectiveness of radiotherapy [37]. There is a synergistic effect when radiotherapy is combined with fluoropyrimidine derivatives (5-fluorouracil, capecitabine and trifluridine/tipiracil), irinotecan and platinum derivatives [38,39]. However, there is a lack of data on the optimal regimen for combining radiotherapy for liver metastases with a specific type of systemic treatment. The only prospective study, RADIOSTEREO-CAMPTO, demonstrated the safety of combining SBRT with irinotecan [40]. The study’s authors indicate that the combination therapy was safe and well tolerated and hypothesise that irinotecan with SBRT can compensate for lower radiotherapy doses (40 Gy in four fractions) due to its radiosensitising effect. The only prospective randomised phase 3 SABR-COMET study found that stereotactic radiotherapy prolonged overall survival (OS) in patients with oligometastatic disease in various cancers without compromising quality of life. However, the group of patients with colorectal cancer in this study was relatively small [41]. It is only possible to indirectly compare standalone systemic treatment in different lines using different drugs with the study group by analysing real-world data. A Spanish study found that median OS and median PFS in the first line were 26.7 and 10.7 months, respectively [42]. The combination with irinotecan resulted in slightly longer treatment times than the combination with oxaliplatin. The anti-EGFR combination also resulted in a longer OS than the anti-VEGFR combination. In contrast, a Dutch study found that median OS in the first line of treatment was 16.3 months [43]. No differences were found between patients treated with the irinotecan or oxaliplatin combination. In the analysed group of patients, median OS and median PFS in the first line of treatment were 27 and 17.5 months, respectively. As in the above studies, a longer OS was observed with anti-EGFR combination therapy than with anti-VEGFR combination therapy, while no differences were found between oxaliplatin and irinotecan combination therapy. In second- and third-line treatment, median OS in real-world data studies was 9–14 months and 6–10.6 months, respectively, while median PFS was 7.6 and 6.2 months, respectively. In our own study, median OS in the second and third lines of treatment was 19 and 13 months, respectively, and median PFS was 11 and 8.5 months, respectively. These data are more favourable than those cited above from real-world clinical practice, suggesting that PFS and OS can be prolonged by adding local treatment to systemic therapy.

The study demonstrated that the combination with radiotherapy was well tolerated. Regardless of the line of systemic treatment used and the type of chemotherapy or targeted therapy, no effect of the combination of brachytherapy with various drugs was demonstrated. The difference in the decrease in albumin levels between patients who did not receive targeted therapy in the second line of treatment and those who received irinotecan in the fifth line of treatment is probably due to the poorer condition of the former group, as this difference is not reflected in other biochemical liver function parameters. The previously cited RADIOSTEREO-CAMPTO study indicates that the combination of hypofractionated radiotherapy and irinotecan chemotherapy is safe. However, studies indicate an increased risk of bleeding with anti-VEGFR therapy (bevacizumab, aflibercept and regorafenib) [44]. Despite the use of brachytherapy alongside anti-VEGFR therapy, no increased risk of bleeding was observed when a minimum interval of two weeks was observed between brachytherapy and anti-VEGFR therapy.

To the authors’ knowledge, this study is the only analysis of this type to date. Despite the uniqueness of the topic, the study has a number of limitations. The main limitation is the retrospective nature of the study, which prevented comparison between patients treated with brachytherapy and those without local treatment and led to the inhomogeneity of the group of patients. Another important limitation is that the choice of systemic treatment for patients was often related to the availability of public funding for specific treatment regimens.

## 5. Conclusions

HDR brachytherapy is a highly effective local treatment for patients with liver metastases when used alongside standard systemic therapy. The study showed that, when patients with *BRAF* mutations or MSI-H/dMMR were excluded, treatment outcomes depend on mutations in the *RAS* genes. Patients without *RAS* gene mutations experience longer PFS, OS, LC and demonstrate a better response to treatment. This is particularly evident in the third and fourth lines of treatment. In the first line of treatment, the effect applies only to PFS; in the second line, it is not observed. The prognosis for patients receiving combination therapy involving brachytherapy also depends on the line of systemic treatment. Median OS, PFS and LC are shorter in subsequent lines of treatment. Tumour reduction is mainly observed in the first three lines of treatment. The data suggest that brachytherapy may be most beneficial when used earlier in the treatment of metastatic colorectal cancer, rather than as a last-line option. The greatest treatment efficacy was observed when brachytherapy was combined with EGFR inhibitors in the first line, and with trifluridine/tipiracil in the third and fourth lines. Combining brachytherapy with chemotherapy or targeted therapy is safe. Regardless of whether targeted therapy or chemotherapy is used, no increase in liver toxicity or risk of bleeding has been observed.

## Figures and Tables

**Table 1:** Characteristics of patients.

Parameter	Number of Patients (Percentage)/Median (Range)					*p*-Value
**Line**—**number of patients (percent)**	1—28 (20%)	2—45 (32%)	3—37 (26%)	4—27 (19%)	5—5 (4%)	
**Age**—**median (range) in years**	64 (32–80)	66 (36–77)	66.5 (35–81)	66 (45–87)	65 (54–74)	0.951
**Sex**						0.440
-Men	10 (36%)	25 (56%)	21 (57%)	13 (48%)	2 (40%)	
-Women	18 (64%)	20 (44%)	16 (43%)	14 (52%)	3 (60%)	
**Tumour localisation**						0.892
-Rectum	18 (64%)	20 (44%)	18 (49%)	11 (41%)	2 (40%)	
-Sigmoid	2 (7%)	9 (20%)	8 (22%)	6 (22%)	1 (20%)	
-Descending colon	4 (14%)	5 (11%)	4 (11%)	4 (15%)	1 (20%)	
-Transverse colon	1 (4%)	4 (9%)	2 (5%)	0 (0%)	0 (0%)	
-Ascending colon	3 (11%)	7 (16%)	5 (14%)	6 (22%)	1 (20%)	
**Number of tumours**						0.029
1	9 (32%)	17 (38%)	11 (29%)	3 (11%)	2 (40%)	
2	10 (36%)	22 (49%)	21 (57%)	10 (37%)	2 (40%)	
3	6 (21%)	5 (11%)	3 (8%)	11 (41%)	0 (0%)	
4	3 (11%)	1 (2%)	2 (5%)	3 (11%)	1 (20%)	
**Volume of all metastases** —**median (range) in cm^3^**	81.3 (1.3–1370)	119.5 (1.5–682.4)	126.1 (1.3–685.4)	90.9 (10.8–1030)	52.3 (4.5–126.2)	0.258
**Metastases outside the liver**						0.219
-No	21(75%)	29 (64%)	23 (62%)	17 (63%)	1 (20%)	
-Yes	7 (25%)	16 (36%)	14 (38%)	10 (37%)	4 (80%)	
**Type of metastases**						0.928
-Synchronic	20 (71%)	33 (73%)	28 (76%)	22 (81%)	3 (80%)	
-Metachronic	8 (29%)	12 (27%)	9 (24%)	5 (19%)	1 (20%)	
***RAS* status:**						0.944
-Wild type	18 (64%)	28 (65%)	25 (71%)	17 (63%)	3 (80%)	
-Mutation	10 (36%)	15 (35%)	10 (29%)	10 (37%)	1 (20%)	
**Intention to treatment:**						0.738
-Repeat oligoprogression	17 (61%)	32 (71%)	22 (59%)	18 (67%)	4 (80%)	
-Induced oligoprogression	11 (39%)	13 (29%)	15 (41%)	9 (33%)	1 (20%)	

**Table 2:** Systemic treatment of patients.

Line	Systemic Treatment	Number of Patients (Percent)
1	Chemotherapy	
	XELIRI/FOLFIRI	13 (54%)
	XELOX/FOLFOX	11 (46%)
	Targeted therapy	
	Anti-EGFR	14 (58%)
	Anti-VEGFR	9 (38%)
	none	1 (4%)
2	Chemotherapy	
	XELIRI/FOLFIRI	21 (49%)
	XELOX/FOLFOX	19 (44%)
	FU/X	3 (7%)
	Targeted therapy	
	Anti-VGFR	27 (63%)
	none	16 (37%)
3	Capecytabine/fluorouracil	12 (32%)
	Trifluridine/Tipiracil	14 (38%)
	Regorafenib	4 (11%)
	Anti-EGFR	7 (19%)
4	Capecytabine/FU-based	
	rechallenge	15 (56%)
	Trifluridine/Tipiracil	4 (15%)
	Anti-EGFR	3 (11%)
	Irinotecan	3 (11%)
5	Capecytabine/FU-based rechallenge	5 (100%)

XELIRI—capecitabine, irinotecan; FOLFIRI—5-fluorouracil, leucovorin, irinotecan; XELOX—capecitabine, oxaliplatin, FOLFOX—5-fluorouracil, leucovorin, oxaliplatin; 5-FU—fluorouracil; X—capecitabine; FU—fluoropyrimidine derivatives (5-fluorouracil, capecitabine); Anti-EGFR—epidermal growth factor receptor inhibitors (cetuximab, panitumumab); Anti-VEGFR—vascular endothelial growth factor inhibitors (Bevacizumab, Afibercept).

**Table 3:** The analysis of the frequency of individual mutations.

Location of Mutation Occurrence	Number of Patients (Percentage)
*K* *RAS*	37 (80%)
Including:	
Exon 2 codon 12 i 13	34 (92%)
Exon 3 codon 61	1 (3%)
Exon 4 codon 117	2 (6%)
*N* *RAS*	9 (20%)
Including:	
Exon 2 codon 12 i 13	4 (44%)
Exon 3 codon 61	5 (56%)

**Table 4:** Impact of treatment lines on 12-month PFS, OS and LC, and median PFS, OS and LC.

Treatment Line	1st Line	2nd Line	3rd Line	4th Line	5th Line	Test/*p*-Value
12-month PFS	77%	53%	29%	0%	0%	44.80/<0.001
Median PFS	17.5 m	11 m	8.5 m	6 m	4 m	
12-month OS	90%	91%	58%	38%	20%	52.42/<0.001
Median OS	27 m	19 m	13 m	11 m	11 m	
12-month LC	92%	89%	32%	24%	0%	41.65/<0.001
Median LC	27 m	19 m	11 m	6 m	6 m	

PFS—progression free survival; OS—overall survival; LC—local control; m—months.

**Table 5:** Test, *p*-value and median survival depending on PFS, OS, LC, percentage decrease in tumour volume and response to RECIST in brachytherapy patients.

Treatment Line		1st	2nd	3rd	4th
PFS	Log-rank test/*p* value	Z = 2.472, *p* = 0.013	Z = 1.780, *p* = 0.075	Z = 2.470, *p* = 0.013	Z = −2.228, *p* = 0.026
	Median *RAS* wt	19 months	13 months	9 months	6 months
	Median *RAS* mt	10 months	10.5 months	5 months	4 months
OS	Log-rank test/*p* value	Z = 1.803, *p* = 0.071	Z = 1.567, *p* = 0.117	Z = 1.994, *p* = 0.046	Z = −2.248, *p* = 0.024
	Median *RAS* wt	27 months	20 months	15 months	11.5 months
	Median *RAS* mt	23 months	17 months	10 months	7 months
LC	Log-rank test/*p* value	Z = 1.372 *p* = 0.170	Z = 1.521 *p* = 0.129	Z = 3.076 *p* = 0.002	Z = −2.51 *p* = 0.012
	Median *RAS* wt	27 months	19 months	11 months	9 months
	Median *RAS* mt	23 months	14 months	8 months	4 months
Percentage decrease in tumour volume	Mann–Whitney U test/*p* value	Z = 0.314, *p* = 0.752	Z = 1.071, *p* = 0.284	Z = −1.923, *p* = 0.054	Z = −1.946, *p* = 0.052
Response to RECIST	Chi square test/*p* value	Chi^2^ = 2.319, *p* = 0.508	Chi^2^ = 5.396, *p* = 0.145	Chi^2^ = 5.736, *p* = 0.057	Chi^2^ = 8.378, *p* = 0.016

PFS—progression free survival; OS—overall survival; LC—local control; RECIST—Response Evaluation Criteria in Solid Tumors; RAS mt.—mutation in the RAS gene; RAS wt—wild-type RAS gene.

**Table 6:** The dependence of liver function biochemical parameters on the type and line of systemic treatment.

Type of Toxicity	Treatment Line	First-Line Chemotherapy	First-Line Targeted Therapy	Second-Line Chemotherapy	Second-Line Targeted Therapy	Third-Line Systemic Treatment	Fourth-Line Systemic Treatment
BIL	Chi^2^ = 12.874	Chi^2^ = 1.360	Chi^2^ = 4.977	Chi^2^ = 3.394	Chi^2^ = 2.027	Chi^2^ = 2.618	Chi^2^ = 0.0
	*p* = 0.116	*p* = 0.503	*p* = 0.289	*p* = 0.494	*p* = 0.362	*p* = 0.855	*p* = 1.0
ALT	Chi^2^ = 14.097	Chi^2^ = 1.360	Chi^2^ = 2.167	Chi^2^ = 2.777	Chi^2^ = 1.469	Chi^2^ = 5.263	Chi^2^ = 2.281
	*p* = 0.079	*p* = 0.506	*p* = 0.705	*p* = 0.595	*p* = 0.479	*p* = 0.510	*p* = 0.892
AST	Chi^2^ = 13.264	Chi^2^ = 1.706	Chi^2^ = 1.040	Chi^2^ = 4.492	Chi^2^ = 1.331	Chi^2^ = 3.4	Chi^2^ = 3.850
	*p* = 0.103	*p* = 0.426	*p* = 0.903	*p* = 0.342	*p* = 0.514	*p* = 0.757	*p* = 0.697
ALB	Chi^2^ = 1.687	Chi^2^ = 0.090	Chi^2^ = 2.0	Chi^2^ = 1.333	Chi^2^ = 6.741	Chi^2^ = 2.916	Chi^2^ = 8.0
	*p* = 0.793	*p* = 0.764	*p* = 0.367	*p* = 0.513	***p* = 0.009**	*p* = 0.405	***p* = 0.046**
PT	Chi^2^ = 2.029	Chi^2^ = 0.010	Chi^2^ = 0.933	Chi^2^ = 0.130	Chi^2^ = 0.035	Chi^2^ = 2.179	Chi^2^ = 3.428
	*p* = 0.730	*p* = 0.919	*p* = 0.627	*p* = 0.936	*p* = 0.850	*p* = 0.536	*p* = 0.330

BIL—total bilirubine; ALT—alanine aminotranspherase; ALT—alanine aminotranspherase; ALB—albumin level; PT—prothrombin time; Chi^2^—Chi square test value.

**Table 7:** The dependence of blood and morphometric parameters, as well as radiological and/or clinical features of bleeding, on the type and line of systemic treatment.

Type of Toxicity	Treatment Line	First-Line Chemotherapy	First-Line Targeted Therapy	Second-Line Chemotherapy	Second-Line Targeted Therapy	Third-Line Systemic Treatment	Fourth-Line Systemic Treatment
HGB	Chi^2^ = 19.251	Chi^2^ = 1.347	Chi^2^ = 1.221	Chi^2^ = 3.057	Chi^2^ = 6.638	Chi^2^ = 4.197	Chi^2^ = 1.934
	*p* = 0.082	*p* = 0.717	*p* = 0.976	*p* = 0.801	*p* = 0.084	*p* = 0.897	*p* = 0.992
PLT	Chi^2^ = 13.877	Chi^2^ = 1.221	Chi^2^ = 0.851	Chi^2^ = 2.144	Chi^2^ = 0.975	Chi^2^ = 1.702	Chi^2^ = 2.657
	*p* = 0.084	*p* = 0.543	*p* = 0.931	*p*= 0.709	*p* = 0.614	*p* = 0.945	*p* = 0.850
bleeding	Chi^2^ = 6.811	Chi^2^ = 0.215	Chi^2^ = 1.387	Chi^2^ = 1.351	Chi^2^ = 1.331	Chi^2^ = 1.105	Chi^2^ = 9.072
	*p* = 0.557	*p* = 0.897	*p* = 0.846	*p* = 0.509	*p* = 0.514	*p* = 0.981	*p* = 0.169

HGB—haemoglobin level; PLT—platelet count; Chi^2^—Chi square test value.

## Data Availability

The raw data supporting the conclusions of this article will be made available by the authors on request.
